# ePatients on YouTube: Analysis of Four Experiences From the Patients' Perspective

**DOI:** 10.2196/med2.2039

**Published:** 2012-04-25

**Authors:** Beni Gómez-Zúñiga, Luis Fernandez-Luque, Modesta Pousada, Eulàlia Hernández-Encuentra, Manuel Armayones

**Affiliations:** ^1^PSINET IN3Universitat Oberta de CatalunyaBarcelonaSpain; ^2^Northern Research InstituteTromsoNorway

**Keywords:** Medical informatics, Internet, patient-physician relationship, health communication, social networks, chronic conditions, YouTube

## Abstract

**Background:**

Many patients share their personal experiences and opinions using online video platforms. These videos are watched by millions of health consumers and health care professionals. Although it has become a popular phenomenon, little is known about patients who share videos online and why they do so.

**Objective:**

We aimed to explore the motivations and challenges faced by patients who share videos about their health and experiences on YouTube. As part of a conference discussion, we asked several patients actively engaged on YouTube to make a video explaining their motivations. This paper discusses these videos.

**Methods:**

In this qualitative study, we performed an analysis of the videos created by 4 patients about their self-reported motivations and challenges they face as YouTube users. First, two judges compared the transcriptions and decided the exact wording when confusing content was found. Second, two judges categorized the content of the videos to identify the major themes.

**Results:**

Four main categories emerged: (1) the origin or cause for making the first video, (2) the objectives that they achieve by continuing to make videos, (3) the perception of community, and (4) the negative consequences of the experience.

**Conclusions:**

The main reason for making videos was to bridge the gap between traditional health information about their diseases and everyday life. The first consequence of sharing their life on YouTube was a loss of privacy. However, they also experienced the positive effects of expressing their feelings, being part of a large community of peers, and helping others to deal with a chronic condition.

## Introduction

Social media are transforming the ways in which people access, create, and use information or services by enabling users to be in contact with others who share their interests or goals. Online videos are popular within social networks, including within the health domain. Currently, more than 500 US hospitals have a channel on YouTube (www.youtube.com) and they have collectively published nearly 50,000 videos [1]. YouTube is not just a repository of videos; it is also a social network where users can interact and socialize (eg, commenting, favorites, and following).

Within the Medicine 2.0 paradigm, health consumers are no longer passive users of health information. Instead, they have organized themselves into online communities where they talk about their conditions and also about their personal experiences [2,3]. Over time, they acquire tacit knowledge about the symptoms of the disease and the effects of medications and also gain pragmatic insights into the realities of adapting to chronic disease [4]. Using information and communication technologies, such as social websites, patients can share their implicit knowledge of their disease. Participating in communities and forums, blogging or tweeting, are some of the activities of the so-called “ePatient,” individuals who are “equipped, enabled, empowered, and engaged in their health and health care decisions” [5].

However, the creation of content by patients is no longer limited to web forums or mailing lists; they now share a wide range of contents (eg, blogs, videos, and photos). Recently, ePatients have started sharing online videos about their health or medical issues [6], which seem to be driven, in part, by the inability of contemporary medical practice to meet the needs of patients that go beyond the traditional treatment they receive, a trait also identified by participants in online health care groups [7]. In addition, on YouTube you can find patient narratives that construct their illness experience, as in the case of a cancer diagnosis [8].

At the 2011 Medicine 2.0 conference, we participated in a discussion about the motivations and challenges facing ePatients when sharing their experiences on YouTube [9]. Four patients participated as co-authors in the presentation through videos in which they discussed their experiences of sharing content on YouTube. The goal of this study is to analyze those videos, with a focus on the psychological perspective of their motivations.

## Methods

### Data Collection

In 2010, Luis Fernandez-Luque invited YouTube ePatients to participate as co-authors in a presentation at the Medicine 2.0 conference [9]. These ePatients can be viewed as key informants. The goal of that presentation was to create a discussion between scholars and ePatients about the challenges and motivations of ePatients on YouTube. Six ePatients with a high number of followers within the diabetes and multiple sclerosis (MS) communities were contacted via their email accounts on YouTube. In total, 4 ePatients agreed to participate and make videos for the Medicine 2.0 discussion. In addition, they agreed to further analysis of the video. An informed consent form was sent to each participant explaining that the videos were going to be shown at a public event and that their speech might be transcribed. As an example, the ePatients were sent a video shown previously at Medicine 2.0 2008 [10,11]. We emphasized that we were seeking their personal experiences; the example was only to help them understand the context of the conference.

Subsequently, these ePatients published their videos on YouTube, thereby making them available to everybody on their YouTube channels. Information about the 4 ePatients is presented in Table 1.

**Table 1:** ePatient information.

YouTube channel name	Age	Sex	Disease	Video link
vbeachy (VB)	47	male	multiple sclerosis	http://www.youtube.com/watch?v=CuOVDAm6tlE
1HappyDiabetic (BW)	31	male	diabetes	http://www.youtube.com/watch?v=jsP9nZXIpME
Laurenvparrott (LP)	29	female	multiple sclerosis	http://www.youtube.com/watch?v=Atq0LP_mfYE
Sixuntilme (KS)	32	female	diabetes	http://www.youtube.com/watch?v=lc9hA4gmlhw

In this study, we analyzed these self-reported motivations and challenges as YouTube users for psychological cues. Although the videos are within the public domain, we asked the key informants for permission to use the content for this study, to which they all gave their consent.

### Video Analysis

A team of psychological experts in eHealth interventions analyzed the videos. The YouTube videos were first transcribed for thematic analyses. During this process, two judges compared and reached a consensus on the transcriptions. The transcribed text was used to perform a content analysis. The coding focused on the aspects related to the motivations and challenges of ePatients on YouTube.

The transcriptions were analyzed independently for themes and patterns. Two judges categorized the video content prior to developing a coding system to interpret the data. Afterwards, the code definitions and categories were refined as new topics emerged in order to identify the major themes. The coding of data was completed with the revised coding.

## Results

We identified four main topic categories in the videos: original motivations, objectives of making videos, social–community factors, and negative aspects. In the following subsections, we address the main results of the analysis for each category.

### Original Motivation

All the key informants reported the origin or their reason for making the videos. One recurrent motivation was being unable to find the online information and guidance they wanted. Research on the Internet led to the ePatients becoming actively involved in creating new resources because these ePatients wanted to bridge the gap between plain information and life with a chronic disease. Thus, a lack of information was the reason why they started creating videos:

BW I was actually not a habitual user of YouTube videos and wasn’t making videos on YouTube before I started doing diabetic videos, but I was actually looking for information and diabetes in a video format and there was nothing there.

Another reason for starting to make YouTube videos for these key informants was to find peers to break their isolation, such as in the following example:

KS ...And part of why I started my blog in the first place was because, even though I’ve lived with diabetes for such a long time and I didn’t known (sic) anyone else who had it, and I literally felt like the only diabetic on the planet.

### Objectives

Their videos provided insight into the reasons why these key informants started to make YouTube videos and, most importantly, why they continued to do so. They described what encouraged them to keep this activity going in four different areas as presented in Table 2.

**Table 2:** ePatients’ objectives in continuing to produce YouTube videos.

Area of encouragement	Representative quote
To help people	“...I think the most important thing that I can do with these videos is help somebody else adjust better with their diabetes...” [BW]
To share my experiences (or mistakes)	“...and I thought that I could share some of these experiences and share some of the mistakes that I’ve made, I might be able to help prevent other people from making some of the same mistakes.” [BW]
To tell my story	“...I’m just telling my story, and I’m glad that encourages and gives inspiration to others who are in a similar situation...” [VB]
To give support for self-management	“So finding others, even in the online space, can make you feel less alone, and feeling less alone can have a huge impact on how you manage your chronic disease.” [KS]

These ePatients wanted to tell other people about their experiences with chronic disease and they also sought explanations for their feelings in certain situations. Moreover, they spoke about the importance of feeling personally supported. This is crucial since they emphasized that sharing feelings (not just information) could improve how they managed their chronic disease. Sharing experiences in a positive light encouraged other patients to make their own decisions and cope with the challenges that come with the disease. As BW explained, it is also important to talk about the mistakes he made because this insight may prevent others from repeating his mistakes. Sharing feelings and experiences impacts the decisions that they make concerning their health.

### Patients’ Community

A common theme across all the videos was the feeling of belonging to a community. We found that one of the most important motivations for these ePatients to continue to make the videos was to find and socialize with others. Across all the videos, knowing that there are other people out there just like them appeared to be very important to the ePatients. In fact, the 4 participants stressed that they had overcome a feeling of being alone (“I literally felt like the only diabetic on the planet”) and now they felt like members of a large community consisting of people from all over the world. That was one of the main benefits of becoming an ePatient and using the Internet.

VB I met so many people from all over the world that I would never have been able to talk to, before the Internet of course, and then now, with the MS community on YouTube it’s incredible.

KS I personally feel supported by my community of other diabetics.

Additionally, key informants reminded us about a fundamental distinction: they were not doctors; they were just telling their stories. They actively pointed out that their videos were addressed to others in similar situations; they produced videos not to give medical advice, but to join a community of peers. A sense of belonging resulted from the responses they received from other patients with the same chronic disease, and made them feel part of other patients’ lives.

LP I take pride in the fact that I make very clear in all my videos that I’m talking about my life, in situations that I’ve been through. And I recommend that people should do their own research, and they should confront their doctor and make the best decision for themselves.

### Negative Effects

Participants also referred to some of the disadvantages or negative consequences that arose from making and sharing their videos. Particularly noteworthy are the loss of privacy, negative or rude feedback, and being targeted to promote specific products.

With regard to privacy, the ePatients emphasized the challenge of sharing personal health information with the world.

BW It’s hard for me to share information about a disease I have, you know. I’m sharing it with the world, literally...any video that I do is shared through the world; it’s just amazing.

Moreover, it is not just strangers who can find out about their symptoms, feelings, or experiences; their friends and relatives also get a closer insight into their disease and what it means in their life. This can be painful for all:

KS Another challenge I face is when...my parents were reading my blog...It’s just kind of made them worry all over again.

Two key informants stressed how deeply affected they were by criticisms or even rude comments made by other Internet users. The ePatients wanted to share their feelings honestly and help other patients. They explained that some users made rude comments and challenged their honesty.

LP They hurt my feelings when I get criticized, because I was in my heart really trying to help other people. So that was really difficult dealing with the criticisms...And I’m absolutely not lying in my videos.

Another ePatient also mentioned this topic, explaining that some users just went to his web to make negative comments and bring negativity.

BW I also receive some negative comments...some really, really rude comments that I have to deal with...I actually have some infiltrators that come to my website as well I share my videos and just start to bring this negativity to diabetes.

Finally, one ePatient explained how some companies wanted to use him to endorse a product or therapy. He described that experience as something negative which could have harmed his reputation.

BW It’s very important not to be a promoter of certain products...I had been solicited by companies that...I really don’t believe in.

## Discussion

Four major themes emerged from the analyses of the ePatients’ YouTube videos: origin, objectives, community, and negative effects. Their main motivation for starting to make the videos was the lack of information—from a patient’s perspective—about everyday aspects of their health conditions. However, their main reason for continuing to share their experience online was to share feelings, support and encourage others dealing with chronic disease, and to make the right decisions. These findings are consistent with the research literature [3,12]. Emotional engagement and sense of agency have been identified as functions of patients’ narratives on YouTube [8]. For our ePatients, sharing their stories made them feel like members of a broad, worldwide community.

However, there are also some drawbacks to making YouTube videos, including loss of privacy and negative feedback and rude comments. Despite those negative consequences, the 4 ePatients felt that they had empowered their peers and had also been empowered by making, posting—and defending—their YouTube videos.

We know that sharing personal experiences about living with a condition has a positive health impact for both listeners and storytellers [13-15]. That may explain why ePatients make their videos despite all the challenges they face. This activity is a clear example of the concept of apomediation defined by Eysenbach [16], since both the production and distribution of videos are done outside health institutions, thereby eliminating professional intermediaries between the producers of the videos and those watching them.

The stories told in the videos confirm that their original motivation came from a desire to add something more “life-related” to treatments followed by patients [8]. In this sense, they created new resources to help others by increasing their involvement in the management of their disease. These statements are consistent with the empowerment of ePatients [5], and also with emerging patient-centric paradigms such as patient-driven research [17].

In addition, the study of the narrative of the ePatient shows that online videos can be a tool that facilitates the interaction between the video producers and those watching them. Social interactions, such as comments, do not just motivate the video creators, but also engage their audience and build an online reputation that increases their visibility. This approach is in contrast to those health channels that avoid communicating with their audience and even disable the commenting function for videos.

There have been many studies on online communities of ePatients analyzing the positive effect they have on their users and empowerment variables [18], self-efficacy, and social support [19-21]. Just as joining an online community has positive effects for ePatients, it is also likely that disseminating videos and posting comments has the same positive effects on the health of patients who say explicitly that they feel they are “building a community” of peers. However, further research is needed to study whether these positive effects are also found in video-sharing communities. Nevertheless, considering empowerment as the result of attitude, dialogue, knowledge, and cooperation processes [21], the recording of, posting, viewing, and commenting on videos may result in more empowered patients. They want to tell their story and to know about other’s stories; they exchange information about the management of their disease; they comment on each other’s posts; and they also create new available resources together.

The videos in this study provide ideas regarding the motivation of patients within online communities. Positive feedback from peers is crucial to sustain their motivation over time. However, negative comments can be very disturbing and decrease their motivation. Therefore, it is very important to provide advice to ePatients who are receiving rude comments and to also establish mechanisms to moderate comments. Patients interested in making videos should be prepared to show a high level of disclosure in order to gain trust from their peers.

With regard to the physician–patient relationship, the key informants in this study said that their experiences were personal and could not be generalized. It is important to mention that their videos were focused on everyday aspects of their diseases, rather than clinical or treatment aspects. In fact, it was their failure to find personal stories about their diseases that motivated them in the first place. They also emphasized that their experiences were not a substitute for medical advice, but rather complementary.

In our opinion, it is crucial that these videos remain personal stories about their own everyday life dealing with their disease. The ePatients studied here tell their own experiences and express their feelings in front of a camera demonstrating an impressive level of self-disclosure. In the research literature, personal stories have been shown to have a positive health impact both on listeners and storytellers [14,15]. Therefore, this type of exercise forms the basis of narrative therapy or even psychodrama used by professionals to treat different problems related to anxiety, depression, or social skills. On the other hand, authors like Kahn [13] found negative correlations between high levels of self-disclosure and anxiety and depression. For personal videos posted by ePatients on YouTube, we believe there is a similar positive emotional effect for both posters and viewers as suggested by Fowler and Cristakis [22] that, in the context of social networks, there is a transmission of dominant emotions.

### Limitations

The main limitation of this study was the very small sample size, as only 4 individuals took part. This limitation also applies to the number of ailments (2): multiple sclerosis and Type 1 diabetes. However, as key informants, these 4 individuals can be considered a suitable sample for obtaining preliminary data. In addition, further studies of YouTube videos and chronic diseases may deepen our understanding of the impact of videos on health consumers. The possible social and motivational impact of comments about the videos also needs to be studied.

Furthermore, we only analyzed one medium (ie, videos) despite the fact that many ePatients combine videos with blogs, personal homepages, and microblogging to construct narratives of their illnesses. This is a clear example of how the social media is fast becoming a ubiquitous communications channel. ePatients do not just share videos, they also use Facebook, Twitter, and/or blogs. That trend makes it very hard to conduct research on the ePatient phenomenon from a holistic point of view.

### Conclusions

The Internet has evolved so that it is not solely a tool to help patients become better informed about their disease or condition and its treatment, but also a platform where some ePatients share their daily experiences of living with their disease with peers. These ePatients make and share videos through YouTube and are creating social networks that go beyond traditional online communities because of their format and ubiquity.

YouTube videos by ePatients are changing our perception of health care and represent a step forward from a perspective centered on the patient to a perspective centered on the patient-to-patient relationship. The patients we studied are empowered from an individual point of view, but also within the community of peers where they are sharing their experiences. As we have seen in the analysis of the videos, the participants feel “surrounded” and accompanied by other patients with the same condition. This new approach has helped improve patients’ daily lives, both for those who watch videos, and for those who make them. In addition, the key informants we studied can also be described as apomediators [23] because they are guiding other health consumers to trusted health information.

Therefore, it is reasonable to assume that YouTube videos by ePatients may help other patients become more involved in the decision making that affects their health and enable them to participate more actively in their health care. In turn, ePatients who express themselves through YouTube videos may also satisfy an emotional need and improve their perception of self-efficacy and empowerment. This is why they continue to make videos and feel good about it.

## Abbreviations

MS: multiple sclerosis
